# Endometriosis mimicking colonic stromal tumor

**DOI:** 10.1093/gastro/gov008

**Published:** 2015-02-26

**Authors:** Vaibhav Wadhwa, Eoin Slattery, Sagar Garud, Saurabh Sethi, Helen Wang, Vitaliy Y. Poylin, Tyler M. Berzin

**Affiliations:** ^1^Department of Internal Medicine, Fairview Hospital, Cleveland Clinic, Cleveland, OH, USA; ^2^Center for Advanced Endoscopy, Division of Gastroenterology, Beth Israel Deaconess Medical Center and Harvard Medical School, Boston, MA, USA; ^3^Department of Pathology, Beth Israel Deaconess Medical Center and Harvard Medical School, Boston, MA, USA; ^4^Department of Surgery, Beth Israel Deaconess Medical Center and Harvard Medical School, Boston, MA, USA

**Keywords:** endometriosis, gastrointestinal stromal tumor, pathology

## Abstract

Endometriosis is defined as the presence of endometrial glands and stroma at extra-uterine sites; it is a common disease affecting women of reproductive age. Endometrial tissue can implant itself to various organs, including the gastrointestinal tract, and can cause significant gastrointestinal symptoms. These ectopic endometrial tissue implants are usually located in the pelvis but can be present almost anywhere in the body. Endometriosis seems to be the most frequent cause of chronic pelvic pain in women of reproductive age and may cause prolonged suffering and disability that negatively affect health-related quality of life. We report a case in a generally healthy young female patient who presented for evaluation of diarrhea.

## Introduction

Endometriosis is defined as the presence of endometrial glands and stroma at extra-uterine sites. It is a common disease affecting women of reproductive age [1]. Endometriosis most frequently causes chronic pelvic pain in women of reproductive age, and may cause long-term symptoms and impaired quality of life [2]. Endometrial tissue can implant to various organs including the gastrointestinal (GI) tract and cause significant GI symptoms. These ectopic endometrial implants are usually located in the pelvis, but can be present almost anywhere in the body.

## Case presentation

**Figure 1.**
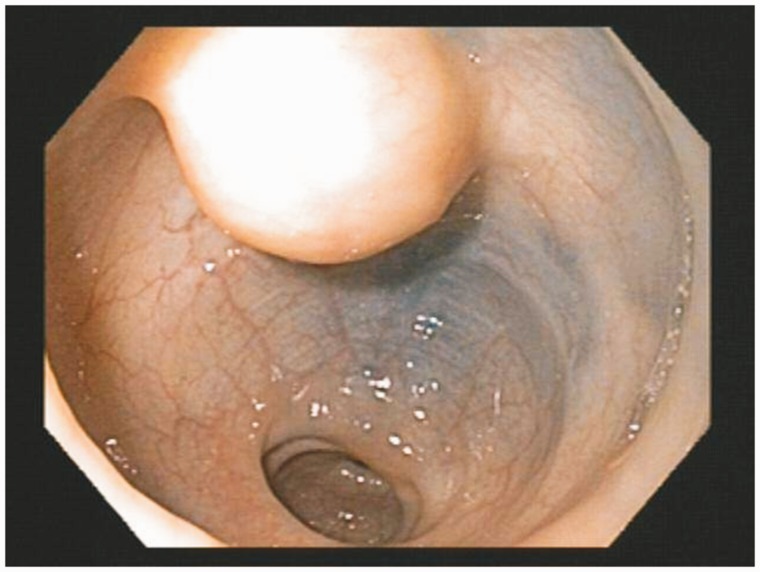
Endoscopic view of the submucosal mass in the sigmoid colon

**Figure 2.**
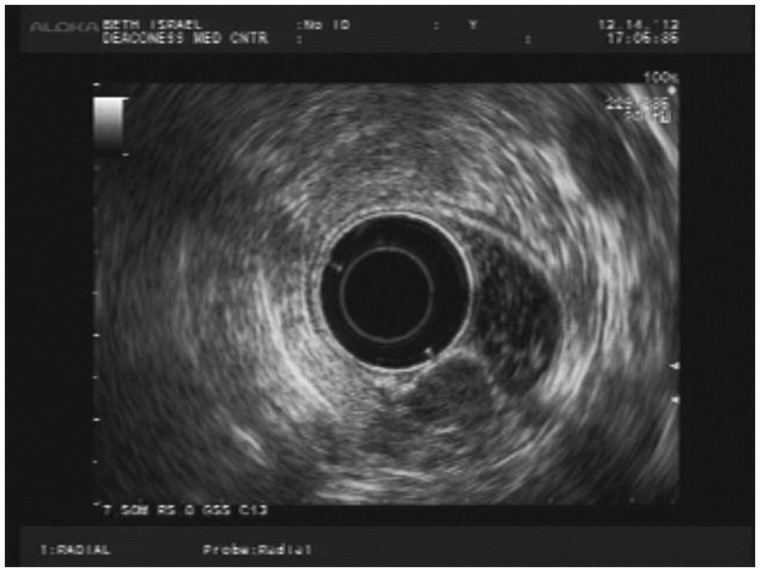
Endoscopic ultrasound image of the mass arising from the *muscularis propria* (EUS layer 4)

**Figure 3.**
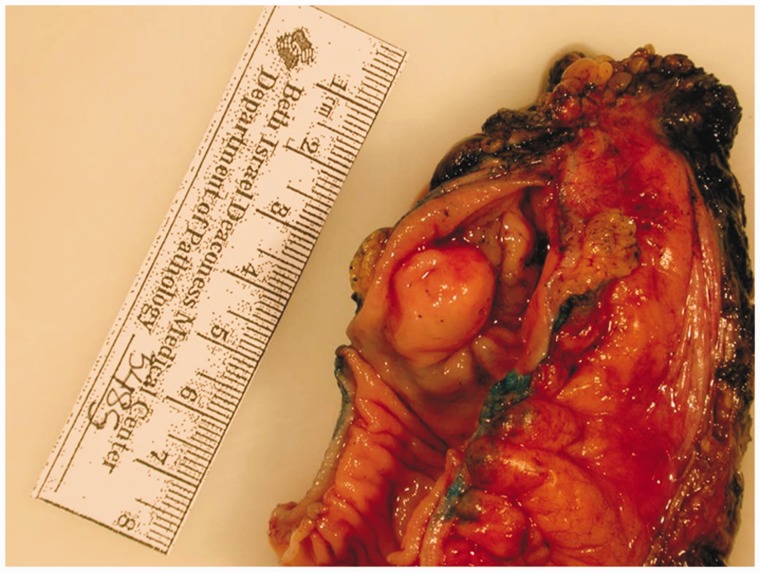
Gross appearance of the surgically resected mass

**Figure 4.**
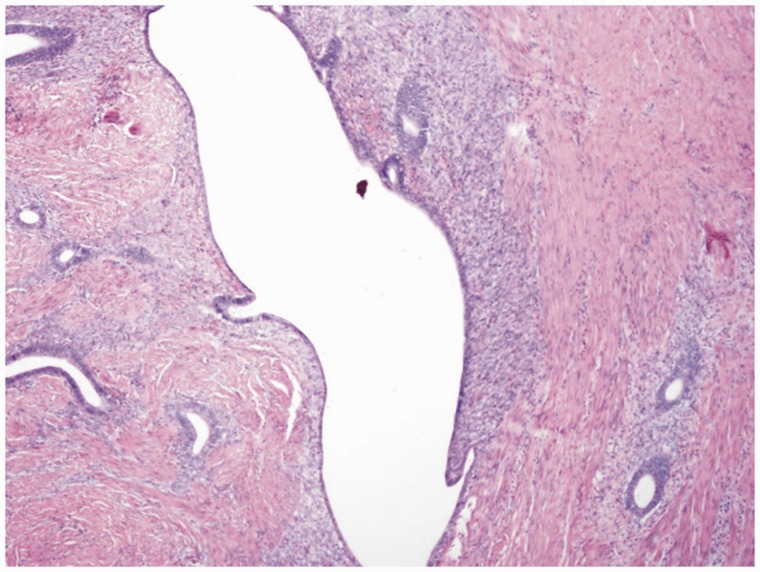
Photomicrograph of sigmoid endometrioma (hematoxylin and eosin stain): lower power view showing endometrial glands and stroma

A 35-year-old female patient was referred to gastroenterology for investigation of a two-week history of self-limiting diarrhea and intermittent left lower quadrant (LLQ) pain. She had a family history of colorectal cancer. Initial physical examination, laboratory investigations and stool cultures were unremarkable. She underwent a sigmoidoscopy, which revealed a submucosal mass in the rectosigmoid colon (Figure 1). The overlying mucosa appeared normal. She was referred to our institution for endoscopic ultrasound (EUS). EUS revealed a round, hypoechoic and homogenous mass measuring 1.5 × 1 cm (Figure 2). The mass was well demarcated and smooth. It appeared to arise from the *muscularis propria* layer (EUS layer 4). Fine-needle aspiration of the mass was performed: the cytology was non-diagnostic. The EUS findings raised concerns that there might be a gastrointestinal stromal tumor (GIST) or leiomyoma; hence she was referred for surgical evaluation. After discussion of the relative merits of clinical surveillance *vs.* resection for presumed GIST, the patient elected to undergo resection. Due to the anatomical location of the mass—approximately 13–15 cm from the rectal verge—transanal minimally invasive surgery (TAMIS) was not feasible. Hence laparoscopic low anterior resection was performed. It was interesting to note that no evidence of endometriosis was found in the pelvis during surgery. Pathological evaluation of the surgical specimen revealed the diagnosis to be endometriosis involving the *muscularis propria* (Figures 3 & 4). Post-operatively, patient’s LLQ discomfort resolved. The diarrhea was later resolved by avoidance of gluten, although evaluation for celiac disease was negative.

## Discussion

Gastrointestinal manifestations of endometriosis are fairly common, occurring in 5–12% of endometriosis patients [3]. The most common locations of the disease in the gastrointestinal tract are the rectum (13–50%), sigmoid colon (18–47%), ileum/small intestine (2–5%) and appendix (3–18%) but it can be present in other locations as well [3–6]. Women with rectovaginal or bowel endometriosis may present with the classic symptoms of endometriosis (dysmenorrhea, dyspareunia, and infertility) and/or with gastrointestinal symptoms. Endometriosis of the bowel wall proximal to the rectosigmoid colon may be associated with non-specific gastrointestinal symptoms. These include diarrhea, constipation, bloating, and abdominal pain [7, 8]. It is also possible that, in certain cases, unrelated gastrointestinal symptoms lead to an endoscopic work-up with the incidental finding of endometriosis, as was probably the case for our patient.

This case is particularly interesting because of the unusual location of the endometrial implant, mimicking a GIST or leiomyoma. Typically in endometriosis of the GI tract, there may be evidence of inwardly penetrating disease from outside the bowel wall. In the case presented, the endometrial implant was confined to the *muscularis propria*, without any involvement of any of the other layers of the colon wall; there was a complete absence of any other endometrial symptoms, which is quite unusual as we see implants and invasion in the setting of significant disease all the time.

The diagnosis of GI endometriosis can be challenging, as the locations of implants and the resulting clinical presentations can vary widely, mimicking numerous other conditions [7].

*Conflict of interest statement:* none declared.
